# Supplementary material for: Serum Folate Concentrations in Exclusively Breastfed Preterm Infants Who Received No Supplementary Oral Folic Acid After Discharge: A Prospective Cohort Study
Source: Nutrients. 2024 Dec 6;16(23):4220. doi: 10.3390/nu16234220 (PMC11644207; doi:10.3390/nu16234220)
Supplement: Supplementary file 1 [file nutrients-16-04220-s001.zip › nutrients-3343531-supplementary.pdf]

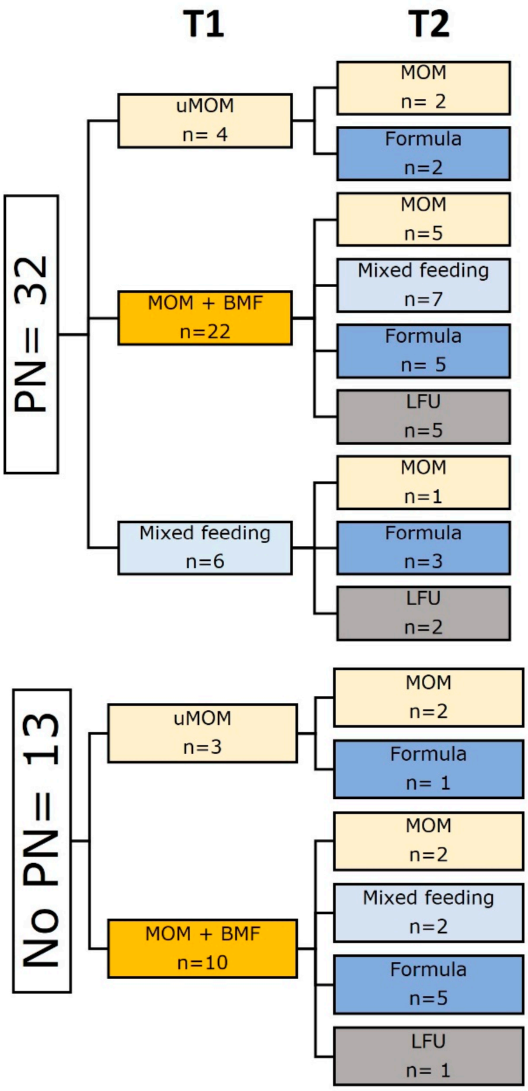

Supplementary Figure S1: Summary of feeding trajectories for participants. A patient was classified as "mixed feeding" if they received any amount of formula. Breast milk fortification was discontinued upon discharge.

PN: Parenteral Nutrition. uMOM: unfortified Mothers' Own Milk. MOM: Mothers' Own Milk. BMF: Breast Milk Fortifier. LFU: Lost to follow up.

Supplementary Table S1: Participant characteristics by type of feeding. Data are median (interquartile range) or n (%).

T1			
	uMOM* (n=7)	MOM + BMF (n=32)	Mixed feeding (n=6)
Estimated added folic acid content of feed (µg/100mL)	0	30	0-30
Gestational age at birth (weeks)	31.4 (30.7-32.3)	29.9 (26.3-31.0)	27.0 (25.8-30.3)
Birthweight (g)	1700 (1575-1871)	1168 (853- 1529)	828 (635- 1405)
Gender: Male	4/7 (57.1%)	13/32 (40.6%)	3/6 (50.0%)
Fetal growth restriction	1/7 (14.3%)	3/32 (9.4%)	0 (0.0%)
Multiple (twin or triplet)	2/7 (28.6%)	12/32 (37.5%)	1/6 (16.7%)
Parenteral nutrition			
Received	4/7 (57.1%)	22/32 (68.8%)	6/6 (100.0%)
Days	5.0 (0.0-7.0)	8.0 (0.0-12.0)	12.0 (8.8- 15.5)
Enteral feeding			
Days at start	1.0 (0.0- 2.0)	2.0 (1.0- 2.0)	3.0 (1.8- 6.2)
Days at full	6.0 (5.0- 8.0)	10.5 (7.2- 13.0)	15.5 (1.5- 23.0)
**T2**			
	MOM (n=12)	Mixed feeding (n=9)	Formula feeding (n=16)
Estimated folate content of feed (µg/100mL)	0	0-58	58
Gestational age at birth (weeks)	30.8 (28.5- 31.5)	30.1 (25.1- 31.6)	29.8 (26.0- 32.1)
Birthweight (g)	1467 (1021- 1827)	1110 (826- 1644)	1235 (823-1564)
Gender: Male	8/12 (33.3%)	6/9 (66.7%)	10/16 (62.5%)
Fetal growth restriction	1/12 (8.4%)	1/9 (11.1%)	1/16 (6.3%)
Multiple (twin or triplet)	0/12 (0.0%)	5/9 (55.6%)	9/16 (56.3%)
Parenteral nutrition			
Received	8/12 (66.7%)	7/9 (77.8%)	10/16 (62.5%)
Days	6.5 (0.0- 9.5)	11.0 (2.5- 15.5)	7.0 (0.0- 12.8)
Enteral feeding			
Days at start	1.0 (0.0- 2.0)	2.0 (1.0- 2.0)	2.0 (1.0- 2.0)
Days at full	8.5 (5.0-12.8)	11.0 (7.5- 20.5)	9.5 (6.5- 15.0)

*Folate content of human milk has been estimated as: weighted mean ± SE (95% CI)): 5.73 ± 0.24 µg/L (5.26 – 6.20) (in a technical report including a metaanalysis of 6 previous studies LASER Analytica, 2014. Comprehensive literature search and review of breast milk composition as preparatory work for the setting of dietary reference values for vitamins and minerals. EFSA supporting publication 2014:EN-629, 154 pp.)
